# Development of celiac-safe foods: prevention of transglutaminase 2 (TG2) deamidation of gluten in healthy non-celiac volunteers

**DOI:** 10.3389/fnut.2024.1308463

**Published:** 2024-03-14

**Authors:** Niklas Engström, Lena Böhn, Axel Josefsson, Stine Störsrud, Nathalie Scheers

**Affiliations:** ^1^Department of Life Sciences, Chalmers University of Technology, Gothenburg, Sweden; ^2^Department of Laboratory Medicine, Lund University, Lund, Sweden; ^3^Department of Molecular and Clinical Medicine, Sahlgrenska Academy, University of Gothenburg, Gothenburg, Sweden

**Keywords:** gluten, celiac, transglutaminase 2, deamidation, ascorbyl palmitate, DGDP

## Abstract

In celiac disease, intestinal transglutaminase (TG2) produces immunogenic peptides by deamidation of gluten proteins. These products drive the celiac immune response. We have previously identified an interaction between gliadin and a food additive, E304i, which prevents gliadin processing (both deamidation and transamidation) by TG2, *in vitro*. In this study, we investigated if E304i could prevent TG2 processing of gluten in flours and if the effect was evident after simulated gastrointestinal digestion. We also confirmed the outcome *in vivo* in a human cross-over intervention study in healthy non-celiac participants. TG2 transamidation experiments (*in vitro*) of digested wheat and rye flours supplemented with E304i at 30 mg/g indicated full prevention of TG2 processing. In the intervention study, participant serum levels of deamidated gliadin peptides (dGDPs) increased after the intake of reference wheat rolls (80 g per day for a week; 41% ± 4% compared to washout), while the intake of the intervention E304i/zinc sulfate wheat rolls generated a modest response (80 g per day for a week; 8 ± 10% of control). The difference between the groups (32.8 ± 15.6%) was significant (*p* = 0.00003, *n* = 9), confirming that E304i /zinc addition to wheat rolls prevented TG2 deamidation of gluten. In conclusion, this study shows that E304i /zinc addition to wheat rolls prevents TG2 deamidation of gluten in non-celiac participants.

**Clinical trial registration:**

clinicaltrials.gov, identifier (NCT06005376).

## 1 Introduction

Celiac disease (gluten intolerance) is an HLA-linked inflammatory autoimmune condition that primarily affects the small intestine. The classical symptoms are pain, diarrhea, and nutrient malabsorption. In genetically pre-disposed individuals, celiac disease may develop in response to a gluten-containing diet. Approximately 98% of the individuals diagnosed with celiac disease possess the combination, or either of, the specific genes HLADQ8 and HLADQ2 (1, 2). Approximately 20–30% of the world population are carriers of these genes, but only 1–2% develop the disease (1–3), indicating that gluten exposure is not the only trigger. The HLADQ8 and HLADQ2 genes are coding for antigen-presenting proteins (HLA antigens) on immune cells, and this particular type presents fragments of gluten-derived deamidated peptides to T-cells (CD4^+^), which triggers the proliferation of gluten-specific T helper (Th1)-cells (4), increasing the sensitivity to gluten-derived deamidated peptides and therefore becoming drivers of the celiac immune response.

Initiation of celiac disease involves intestinal tissue transglutaminase (TG2; E.C. 2.3.2.13) (5–7). The key to the initiation of an (auto-) immune response toward gluten is the production of deamidated peptides, which are catalyzed by TG2 (5). TG2 binds specifically to a binding motive (gln-X-pro) on gluten peptides and catalyzes the transamidation of the peptides in the presence of a primary amine and the deamidation of the peptides in the presence of water. The deamidation reaction produces negatively charged peptides, converting glutamine (gln) to glutamic acid (glu) residues. These deamidated peptides are relatively long-lived, and consequently, the level in the intestinal wall becomes high after dietary gluten exposure. The activity of TG2 is regulated by the trace metal ions calcium (Ca^2+^) and zinc (Zn^2+^), whereas Ca^2+^ promotes and Zn^2+^ inhibits its activity (8–10). Therefore, the dietary levels of calcium and zinc may also affect the intestinal level of deamidated peptides after a gluten-containing meal. Celiac disease is an autoimmune disease in which the immune system attacks TG2 by producing autoantibodies toward the enzyme. The immune system-assisted removal of TG2 (TG2 autoantibodies) is associated with the upregulation of TG2 in the small intestine in active celiac disease (7), further escalating the progression of the disease. Antibodies toward transglutaminases (TG) are thus sensitive diagnostic markers for celiac disease. A diagnosis of celiac disease can be made in young children based on serology alone without a confirmatory biopsy if the anti-TG titer is high (11).

The long-term goal of our studies is to provide the tools to facilitate the production of flours and wheat rolls with TG2-inaccessible gluten-binding motives. Previously, we computationally screened approximately 5 million molecules for their affinity toward the most common TG2-binding motive, gln-lys-pro, in alpha-gliadin (12), from which 18 molecules were selected for TG2 transamidation experiments. The experiments showed that one of these molecules, the food additive ascorbyl palmitate (E304i), reduced transamidation of gliadin (by 80% at 15 μM). To completely prevent the transamidation, we conducted dose–response experiments with E304i and gliadin in addition to adding a zinc salt (zinc chloride) to reduce the TG2 intrinsic activity. The degree of deamidation of gliadin was measured by an antibody toward deamidated gliadin peptides (dGDPs), which confirmed that there was no significant deamidation of gliadin in the presence of E304i or E304i with zinc chloride. The rationale for measuring *trans*amidation, and not *de*amidation, for estimating TG2 binding (hence processing) to gluten peptides is a methodological consideration since the primary amine tagged with biotin can efficiently be used for detection purposes. Water molecules taking part in the deamidation reaction would be difficult to discern from all the other water molecules in the solution.

In the present study, the aims were (1) to estimate the ratio of E304i/to flour for full prevention of TG2 binding, (2) to conduct pilot experiments to evaluate the taste of wheat rolls containing those amounts of E304i, and (3) to evaluate the *in vitro* data in a human intervention study in healthy participants. First, we estimated TG2 binding to gluten peptides in wheat and rye flours after a simulated gastrointestinal digestion step by running human TG2 transamidation assays to determine the amount (mg) of E304i per g of flour needed to prevent TG2 binding. This information was used to make breakfast wheat rolls that were used in this blinded pilot study (*n* = 2 participants) to evaluate if the participants could taste the E304i/zinc additive in the wheat rolls. Finally, a randomized double-blind cross-over intervention in healthy participants was conducted, in which the endpoint was dGDP measured in participant blood, to determine if the ingestion of wheat rolls containing the E304i/zinc additive would lead to less or no TG2 processing of gliadin in comparison to reference wheat rolls (no additives).

## 2 Materials and methods

### 2.1 Flours

A few different wheat and rye flour types, both whole grain and endosperm flours, were included in the pre-studies. All flours were produced in Sweden by Nord Mills and provided by Lantmännen Cerealia Ltd., Malmö, Sweden (Table 1).

**Table 1:** Types of flours used in the pre-studies and their protein and fat content.

Flours	Protein (g/100 g)	Fats (g/100 g)
Sifted spelt: spelt (wheat) flour and malted wheat flour	13	1.5
Wheat: 100% endosperm wheat flour	11	1.6
Biscuit flour: Wheat flour ground on 100% autumn wheat and malted wheat flour	9.5	0.4
Whole grain wheat: fine ground whole grain wheat 100% (endosperm, hull, and husk).	11	2.2
Whole grain rye: Wholegrain rye (endosperm, hull, and husk) and malted barley flour.	7.7	1.9
Sifted rye: Rye flour and malted barley flour.	7.0	1.5

### 2.2 Determination of gluten content in wheat and rye flours

A commercially available assay to estimate gluten content (AgraQuant® ELISA gluten G12; Romer Labs) was chosen since the assay antibody (G12) is specific to the TG2-binding motive QLP, which our studies specifically focus on. The assay was performed according to the manufacturer’s instructions. After data analysis, we calculated the theoretical content of E304i (per gram of flours) estimated to be needed to fully cover all TG2 binding sites in the wheat and rye flours.

### 2.3 Simulated gastrointestinal digestion of flour samples

Wheat and rye flour samples went through a simulated gastrointestinal digestion (*in vitro*) according to a simplified standard protocol used in our laboratory, with modifications (13). First, flour samples (1 g) were suspended in water (10 mL) and digested with a pepsin solution containing 0.16 mg (2,500 U/mg)/L of HCl (0.1 mM) at pH 2 for 1 h at 37°C. Then, the pH was adjusted to 7 by the addition of NaHCO_3_ (1 M). The samples were further digested with the pancreatin solution (1.7 mL/sample containing pancreatin; 4 g/L) for 1 h at 37°C. After the digestion, the digests were cooled down to inhibit further digestion.

### 2.4 TG2 transamidation assay

To estimate TG2 binding to gluten peptides in full flours, we have developed a transamidation assay based on the method of Skovbjerg et al. (14). Briefly, 96-well plates were coated overnight at 4°C on a rotary shaker at 20 rpm, with freshly digested flour samples, with and without E304i at 5, 10, and 30 mg/g flour. The wells were washed three times with TBS (5 mM) + Tween (0.05%) and blocked for 1 h at room temperature using Sigma’s coating stabilizer and blocking buffer (# C9483, Sigma Aldrich, St. Louis, MO, US). A biotin solution, containing 5-(biotinamido) pentylamine (0.165 mg/mL) mixed with Tris–HCl (5 mM), CaCl_2_ (5 mM), and dithiothreitol (10 mM), mixed with TG2 (0.5 U/mL, human recombinant; #T022, Zedira, Darmstadt, Germany), was made fresh and added to each well to incubate for 1 h at 37°C and 30 rpm. The plate was then washed and europium-streptavidin (1 μg/mL, DELFIA; PerkinElmer, Waltham, MA, US) in DELFIA assay buffer (PerkinElmer) was added, and then the plate was incubated for 1 h in room temperature. After another washing, Delfia enhancement buffer (PerkinElmer) was added, and the plate was incubated for 5 min, after which the fluorescence (345 nm excitation, 617 nm emission) was measured. The reduction in transamidation by E304i was compared to untreated flour controls and presented as % of control flours.

### 2.5 A randomized double-blind intervention study in healthy volunteers

#### 2.5.1 Study design

**Figure 1:**
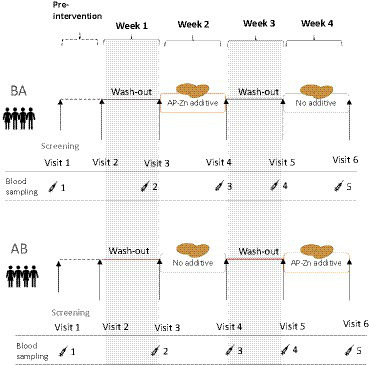
Overview of the randomized cross-over study design. The group (BA) started with the wheat rolls containing the E304i/zinc additive and the group (AB) started with the reference wheat rolls.

The study was a randomized, double-blind, 4-week human dietary intervention with a cross-over design in healthy participants. Celiac disease and other diagnosed enteropathies were among the exclusion criteria. It was also a requirement that the participants eat gluten products as part of their habitual diet (to avoid asymptomatic celiacs and to promote compliance). The endpoint was serum-dGDP as an indicator of TG2 deamidation of gluten peptides in the intestinal wall. The 4-week intervention started with a wash-out week (gluten-free diet) to reduce participant blood levels of dGDP. The participants were instructed to not eat gluten-containing foods, except for the intervention/reference bread rolls, throughout the whole study. The participants met the research staff at the screening and once a week during the intervention periods. At the weekly meetings, the participants were provided with gluten-free staples such as pasta, rice, and bread, in addition to the intervention bread rolls. The nutritionist/dietician followed up on the previous week’s food diary and the participants’ wellbeing/health status. The study design is outlined in Figure 1.

#### 2.5.2 Participants

**Figure 2:**
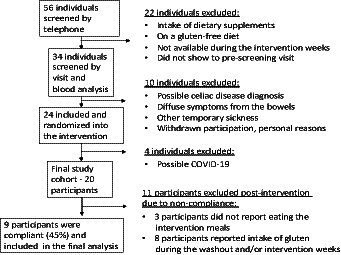
Inclusion/exclusion scheme.

A total of 56 individuals of both genders between the ages of 18 and 50 years (BMI 18.5–32) were pre-screened in the first round (on the phone). Of the 56 individuals, 34 went through the screening process (a visit + blood analysis), and then, 24 were invited to participate in the study and block-randomized (starting with A or B) into each intervention arm (BA or AB). Out of the 24 who were invited, four were excluded during the intervention. Reasons for the late exclusion were the common cold/possible coronavirus infection. A total of 20 participants went through the full intervention study, and after exclusion due to non-compliance, 9 participants remained in the end (Figure 2).

#### 2.5.3 The intervention meal

The intervention meal (breakfast) consisted of two wheat rolls (á 42 g) with poppy seeds. The purpose of the poppy seeds was to cover differences in the appearance of the crust to ensure the blinding of the study. Standard recipe wheat rolls were baked at Lantmännen Unibake’s production site in Finland. One reference dough and one dough supplemented with E304i (3% of flour weight) and zinc sulfate heptahydrate (0.0125% zinc of dough weight) were produced. The wheat rolls were packaged in color-coded bags, frozen, and transported to Sweden. The identity of the color codes was not known to the personnel in contact with the participants. The cold chain was not broken until the weekly ration had reached the participants. The instructions were that the breakfast rolls could be prepared with any gluten-free spread of the participant’s own choice.

#### 2.5.4 E304i addition

E304i, ascorbyl palmitate, is generally recognized as safe (GRAS) and approved by the US Food and Drug Administration (USFDA) as a food additive. It is frequently used as an antioxidant or as a fat-soluble form of vitamin C. The European Food Safety Authority (EFSA) is in line with the American authorities and has also concluded that there is no safety concern regarding using E304i as a food additive (15). According to EFSA, E304i is considered to have the same biological effects as ascorbic acid, and from human data, it was concluded that supplementary doses of approximately 1 g/person per day in addition to normal dietary intake are not associated with adverse gastrointestinal effects, but gastrointestinal acute effects may occur at doses exceeding 3–4 g/day. One intervention wheat roll (42 g baked roll) contained 0.84 g of E304i and the intake of two of these rolls per day contained 1.6 g of ascorbyl palmitate/participant/day.

#### 2.5.5 Addition of a zinc salt

Zinc chloride was used as the zinc salt in the pre-clinical studies, and zinc sulfate was the choice for the intervention wheat rolls in the human study. Zinc sulfate is the compound normally used for the zinc fortification of foods. The recommended daily intake of zinc is 11 mg/day for men and 8 mg/day for women in the US (16). However, the intestinal absorption of zinc from the diet has been reported to be 15–60%. Dietary zinc supplements come in daily doses in the range of 10–50 mg, and a common supplemental dose is approximately 30 mg/day. An upper tolerable level for zinc has been estimated at 40 mg/day for adults and 12 mg/day for children in the age range of 4–8 years old in the U.S. (16). Symptoms of mildly overdosing zinc were mainly related to gastrointestinal discomfort, while higher doses negatively affected body copper balance. Zinc salts such as zinc chloride, zinc acetate, and zinc sulfate are approved within the E.U. as dietary zinc supplements. The intervention wheat rolls (per 42 g of baked roll) contained 6.2 mg of zinc per roll, leading to an intake of 12.4 mg of zinc per day during the intervention study.

#### 2.5.6 Biological samples

Blood samples were collected (heparin and serum tubes) during the study visits by nurses at the Gothia Clinical Trial Center (CTC) at Sahlgrenska University Hospital, Goteborg, Sweden. Samples were kept on ice, immediately processed, and aliquoted. All samples were stored at −20°C during the day of collection and then transferred to −80°C freezers at Chalmers University of Technology, Goteborg, Sweden. Samples were analyzed for dGDP with ELISA (primary antibody ab36729; Abcam).

#### 2.5.7 Statistical analysis

Basic statistical analyses were performed by Microsoft® Excel for Mac (version 16.67). Two-tailed T-tests were performed based on unequal (2,3) or equal variances (2,2). The Sdev.P function was used to estimate the standard deviation at the population level and the sdev for population samples. To our knowledge, there are no previously published data on the serum levels of deamidated gliadin peptides since the measure is normally *antibodies* to deamidated gliadin in celiac patients or healthy individuals. Therefore, the assumptions for the sample size calculations were made based on preliminary measurements in our laboratory. We used an online tool to estimate sample size for a cross-over study where the outcome is a measurement.^1^ A total of 40 participants were to enter the study, assuming a power of 80%, a two-sided significance level of 5%, and a potential drop-out rate of 18%. After the study, the power was recalculated using the achieved results, and it was estimated that the power based on the compliant participants, *n* = 9, was 90% (5% two-sided significance). Thus, the original calculations greatly underestimated the difference between the groups. The randomization was performed using an online tool.^2^

## 3 Results

### 3.1 Gluten content

**Figure 3:**
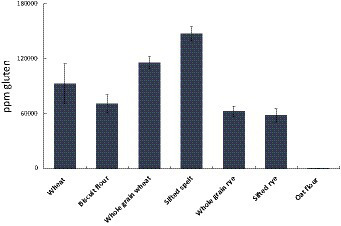
PPM Gluten per gram flour (or PPM QPQLPY, in which QLP is one (out of seven) TG2-binding motives). The results indicate: Wheat >Rye. Below 20 PPM is regarded as “gluten free”.

The AgraQuant® assay was developed by Romer Labs to measure gluten content. The method builds on an antibody (G12) that binds one immunogenic amino acid sequence in wheat (QLP), in which QLP is one of the binding motives of TG2. We calculated the theoretical content of TG2 binding sites to roughly estimate the amount of E304i needed to cover all TG2 binding sites in the flours (Table 2). The relative levels of experimentally estimated gluten (with the G12 assay) correlated with the number of QXP motives and calculated amount of E304i to cover them (Table 2 and Figure 3).

**Table 2:** Theoretical amounts of E304i to cover the QXP binding sites (mg/g flour).

Flours	E304i (mg/g flour)
Wheat	21,85
Biscuit flour	16,78
Whole grain wheat	27,39
Sifted spelt	34,75
Whole grain rye	19,47
Sifted rye	18,03

### 3.2 TG2 processing of wheat and rye flours

**Figure 4:**
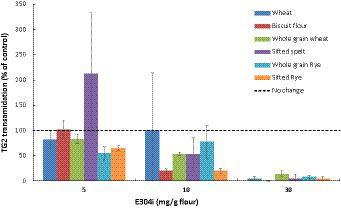
Dose-dependent decrease in the human TG2 transamidation of wheat and rye flours at different concentrations of the food additive E304i. The supplemented flours were exposed to simulated gastrointestinal digestion. Data are presented as a percentage of control (0 mg E304i/g flours) and are means ± Sdev, *n* = 3 experiments. The TG2 transamidation at 30 mg E304i/g flour was not significantly different from the control (*p* > 0.5).

Dose–response experiments with three different concentrations of E304i (5, 10, and 30 mg E304i /g flour; *n* = 3) were conducted. A measure of 30 mg E304i/g flour was required to fully prevent TG2 processing of wheat and rye flours, as anticipated by the theoretical estimations (Figure 4). The biscuit flour, which showed the highest content of TG2 processing of the untreated refined wheat flours (except for the sifted spelt flour), showed a marked decrease in TG2 processing already at low E304i levels (10 mg/g flour, 71% decrease, with no addition of zinc). The TG2 activity in both wheat and rye flours was not sufficiently lowered at 10 mg E304i /g flour. TG2 processing of gluten by all the flours at 30 mg E304i/ flour was not significantly different from baseline, indicating a full prevention of TG2 processing. This finding was the rationale for choosing the dosage of 30 mg E304i/g flour in the human intervention study.

### 3.3 Perceived taste of bread rolls with added E304i/zinc sulfate-a pilot study

A pilot study (*n* = 2) to evaluate if the E304i addition to a wheat roll would cause sensory issues was undertaken. The results (data not shown) indicated that there was no difference in taste between wheat rolls containing E304i or E304i/zinc sulfate compared to the control wheat roll with no additives. The blindfolded participants could not identify which roll contained E304i/zinc sulfate. However, the E304i additive was observed to be sensitive to high heat (200°C oven temperature) since the crust appeared with tiny dark spots. These spots were only visible on the crust. Therefore, we added poppy seeds to the intervention bread rolls to facilitate the blinding of the study.

### 3.4 The E304i/zinc sulfate bread roll intervention study

#### 3.4.1 Serum levels of dGDP

Participant blood levels of dGDP were measured in serum samples taken at baseline and then after each washout and intervention week (a total of five sampling events, Figure 1). From our studies, we learned that the serum levels of dGDP were easily affected by the ingestion of gluten, with the consequence that non-compliance or intake of trace amounts of gluten-containing foods had large effects on the data. Non-compliance was counted as (1) no reported intake of the intervention/reference bread rolls and (2) reported intake of gluten-containing foods (in addition to the intervention/reference wheat rolls). Baseline levels of dGDP in participants after washout differed by a factor of approximately 2.5 and, on average, were 422 ng dGDP/ml serum among the participants who remained at the end of the study (*n* = 9). In a pilot pre-study before the intervention, we estimated dGDP levels in serum from an individual on a habitual gluten-free diet (non-celiac) to 28.2 ng dGDP/ml serum, and after an arbitrary gluten intake for 2 weeks, the serum concentration of dGDP was 106.9 ng/mL, indicating that the dGDP levels are sensitive to gluten exposure and may be a candidate (compliance) biomarker for gluten intake (here used as a marker for deamidation of gluten by TG2).

**Figure 5:**
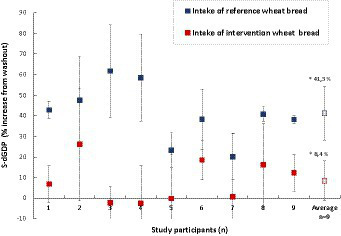
Changes in participant (*n* = 9) serum levels of dGDP after the intake of reference wheat bread rolls (blue marker) and after the intake of E304i/zinc sulfate-supplemented wheat rolls (red marker). The average change at the group level was 32.8 ± 15.6% (*n* = 9, *p* = 0.00003). The asterisks indicate a significant difference between the reference and intervention groups.

The outcome of the study was based on nine individuals (*n* = 9), of whom six participants (*n* = 6) belonged to the AB group and three (*n* = 3) to the BA group (where bread A was the reference roll and B the E304i/zinc sulfate-added wheat roll). The serum levels of dGDP in response to the E304i/zinc sulfate-added wheat roll and the reference roll, respectively, were normalized to each participant’s dGDP status before the intervention week (washout levels). The percentage change within each participant was then compared at the group level. The difference in participant serum-dGDP after 1 week of having two reference wheat rolls per day (gluten exposure) in comparison to having the E304i/zinc-added wheat rolls was 32.8% ± 15.7% (*p* = 0.00003) on the group level (statistical power 90%, with the result explained in Section 2.6), Figure 5. The increase in participant serum-dGDP after the E304i/zinc-added wheat rolls vs. washout was an average of 8 ± 10% and after the intake of the reference wheat rolls, 41% ± 4% vs. washout.

## 4 Discussion

The approach taken in this study is to prevent intestinal TG2 from binding to gluten peptides by adding the food additive E304i, which we have previously found to interact strongly with TG2 binding motives on gliadin. In this way, the catalysis of both transamidation and deamidation reactions was prevented. Therefore, we can assess either the transamidation or deamidation reactions to prove if the binding of TG2 was hindered by E304i. However, it is the deamidation reaction that initiates the celiac immune response. In studies by Italian researchers, an approach based on the same starting point was used, in which they used a microbial TG to crosslink a primary amine to gliadin extracted from wheat flour, which was observed to block the production of the cytokine interferon **γ** (IFN- **γ**) in gluten-restricted T-cells (17). In the same study, the results suggested that transamidated gliadin could still bind the DQ2 surface protein on antigen-presenting cells. Some years later, the research group conducted a human intervention trial with celiac patients on the concept and found that as many as 37% (on day 15) in the group challenged with transamidated flours experienced clinical relapse, while the relapse in the control group challenged with non-deamidated flours was 75% (18). However, with somewhat promising results, the question should be whether or not transamidated gliadin peptides could be immunogenic as well, but to lesser extent than deamidated intermediaries.

In the present study, the highest concentration of the TG2 binding motive blocker E304i was limited to 30 mg E304i /g flour to be suitable for human ingestion. At this level (30 mg/g flour), two bread rolls can be ingested daily, without exceeding the recommended daily allowance (RDA). Even higher levels have been reported in the literature, and the RDA differs in the EU and the US, suggesting that there seems to be no real toxicity risk. Approximately 80% of E304i seems to be retained after heating, and the breakdown products are unharmful (ascorbic acid and palmitic acid), as reported by EFSA (19). The prevention of transamidation was predicted to decrease further with the addition of a zinc salt to inhibit the intrinsic activity of TG2, as was observed with pure gliadin in our previous studies (12). The compliance of the study participants was 45% (*n* = 9 individuals) out of the 20 individuals who finalized to participate in the study. Among the compliant individuals, it was sometimes evident that there was an increased basal level of dGDP, suggesting that the participants were still exposed to unreported gluten. This was not considered as non-compliance since the participants were not instructed to keep a strict gluten-free diet, only to avoid gluten-containing foods such as wheat-based pasta and bread. The outcome (through lower baseline dGDP) would have benefited from the participants being on a strict gluten-free diet (except for the intervention wheat rolls), and this was a weakness of the study.

The study also indicated that the difference between the intervention groups would be greater if the washout periods were considerably longer. The short washout periods were, therefore, another weakness of the study. A long-term study with participants on gluten-free diets with the E304i /zinc-added wheat rolls as the only gluten exposure would have been ideal. However, in conclusion, this study shows that E304i /zinc addition to wheat rolls prevents TG2 deamidation of gluten in non-celiac participants.

## Data availability statement

The raw data supporting the conclusions of this article will be made available by the authors, without undue reservation.

## Ethics statement

The studies involving humans were approved by the Swedish ethical review authority (#2020-00673). The studies were conducted in accordance with the local legislation and institutional requirements. The participants provided their written informed consent to participate in this study.

## Author contributions

NE: Formal analysis, Investigation, Methodology, Validation, Visualization, Writing – review & editing. LB: Investigation, Writing – review & editing. AJ: Investigation, Writing – review & editing. SS: Writing – review & editing. NS: Writing – review & editing, Conceptualization, Data curation, Formal analysis, Funding acquisition, Methodology, Project administration, Supervision, Validation, Visualization, Writing – original draft.

## Footnotes

^1^ hedwig.mgh.harvard.edu/sample_size/js/js_crossover_quant.html

^2^ sealedenvelope.com/simple-randomiser/v1/lists
